# Cassava Witches’ Broom Disease in Southeast Asia: A Review of Its Distribution and Associated Symptoms

**DOI:** 10.3390/plants12112217

**Published:** 2023-06-04

**Authors:** Juan M. Pardo, Khonesavanh Chittarath, Pinkham Vongphachanh, Le Thi Hang, Samoul Oeurn, Warren Arinaitwe, Rafael Rodriguez, Sok Sophearith, Al Imran Malik, Wilmer J. Cuellar

**Affiliations:** 1 Cassava Program, International Center for Tropical Agriculture (CIAT), The Americas Hub, Km 17 Recta Cali-Palmira, Cali 763537, Colombia; 2 Plant Protection Center (PPC), Department of Agriculture, Ministry of Agriculture and Forestry, Vientiane P.O. Box 811, Laos; 3 Plant Protection Research Institute (PPRI), Duc Thang, Bac Tu Liem, Ha Noi 100000, Vietnam; 4 Plant Protection Sanitary and Phytosanitary Department, General Directorate of Agriculture (GDA), Phnom Penh 120406, Cambodia; 5 Cassava Program Asia Office, International Center for Tropical Agriculture (CIAT), Vientiane P.O. Box 783, Laos; 6 Cassava Program Cambodia Office, International Center for Tropical Agriculture (CIAT), Phnom Penh 120904, Cambodia

**Keywords:** cassava witches’ broom disease, phytoplasma, phyllody, diagnostics

## Abstract

Cassava witches’ broom disease (CWBD) is one of the main diseases of cassava in Southeast Asia (SEA). Affected cassava plants show reduced internodal length and proliferation of leaves (phyllody) in the middle and top part of the plant, which results in reduced root yields of 50% or more. It is thought to be caused by phytoplasma; however, despite its widespread distribution in SEA still little is known about CWBD pathology. The overarching goal of this study was to review and corroborate published information on CWBD biology and epidemiology considering recent field observations. We report the following: (1) CWBD symptoms are conserved and persistent in SEA and are distinct from what has been reported as witches’ broom in Argentina and Brazil. (2) In comparison with cassava mosaic disease, another major disease of cassava in SEA, symptoms of CWBD develop later. (3) Phytoplasma detected in CWBD-affected plants belong to different ribosomal groups and there is no association study available indicating phytoplasma as the causing agent of CWBD. These findings are essential clues for designing surveillance and management strategies and for future studies to better understand the biology, tissue localization and spatial spread of CWBD in SEA and other potential risk areas.

## 1. Introduction

Cassava witches’ broom (CWBD) is a devastating disease of cassava (*Manihot esculenta* Crantz) in Southeast Asia (SEA). Throughout this region, cassava is recognized as a prominent food security crop for poor and vulnerable communities. As an example, over the past 20 years, the cultivated area of cassava in Cambodia has increased by >15 times, from 19,600 ha in 2002 to approximately 388,000 ha in 2018. Cassava is now considered the second most important crop after rice and largely surpasses maize in upland production systems in SEA [1].

There were virtually no official cassava phytosanitary constraints reported in the region before 2008. The mealybug *Phenacoccus manihoti*, not known to occur in Thailand until that year [2], soon spread to neighboring countries [3], followed by CWBD, which reached the highest reported incidences in 2014 [4] (See below). Then, the Sri Lankan cassava mosaic virus, causing cassava mosaic disease (CMD), emerged and spread rapidly in the whole region from 2015 onwards [5]. With cassava being a vegetatively propagated crop, dissemination of diseases does not require specific vectors; instead, dissemination of a disease in the region is facilitated by the inadvertent distribution of contaminated material via official and unofficial seed networks [6]. 

While CWBD remained largely unnoticed in this region, in 2010, more than 60,000 ha in the provinces of Yen Bai, Quang Ngai and Dong Nai located in north, central and south Vietnam, respectively, were reported as severely affected by this disease, with incidences as high as 80%, and reductions in root yield and starch content of 30% [7]. In 2012, cassava farmers in Cambodia reported yield losses of up to 50% in the provinces of Kampong Cham, Kratie and Prey Veng, close to the southern border with Vietnam. CIAT’s cassava pathology team working in collaboration with colleagues in the region reported that over 80% of the surveyed cassava fields were affected at infection rates of 35–40% in this country [8]. Around this time, field surveys carried out in the cassava growing provinces in Chachoengsao and Rayong in southern Thailand evidenced a high incidence of the disease, associated with significant yield losses and reduced starch content in plants showing distinct symptoms of CWBD [9]. In all these cases, sequences of a *Candidatus* phytoplasma of the asteris group were identified in leaf samples from affected plants (see below).

To the best of our knowledge, CWBD has only been officially reported in SEA and appears to be an endemic disease of cassava in this region, whose management has been hindered by a lack of knowledge on the causal agent. Our goal here was to review and corroborate published information on CWBD biology and epidemiology considering recent field observations. We provide some insights for designing surveillance and management strategies. Future studies to identify resistant varieties, and understand the infection and transmission of CWBD in SEA and other potential risk areas, will require the identification of the causal agent of the disease.

## 2. Symptoms and Pathogens Identified in CWBD-Affected Plants

**Figure 1:**
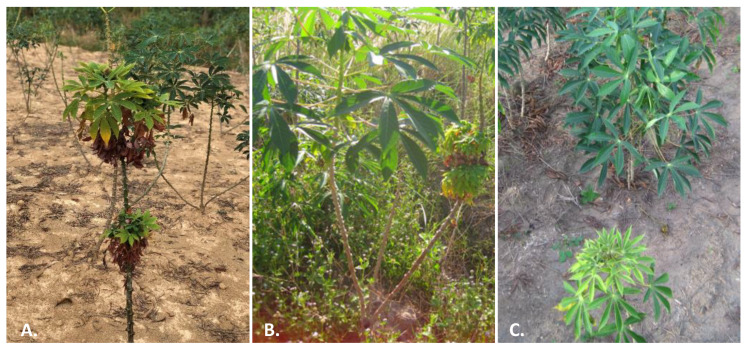
Cassava witches’ broom symptoms. (**A**) Twelve months old cassava plant with symptoms of little yellow leaves, sprout proliferation on the middle and top parts and short petioles. (**B**) Seven months old plant showing typical symptoms only in one of the stems (right) of a cassava plant. (**C**) Early symptoms in young plants. Upper plant is healthy; bottom plant shows dwarfism and leaf yellowing.

**Figure 2:**
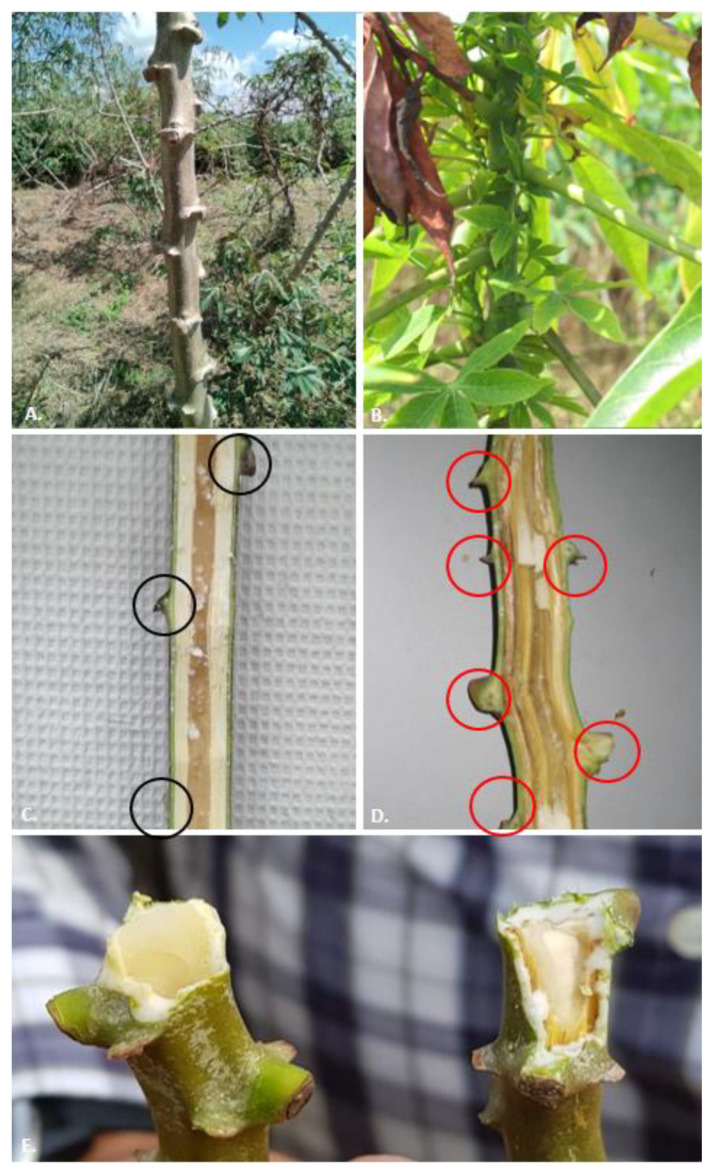
Lignified cassava stems. (**A**) Cassava stem with normal internodes, without any sprout along the stem. (**B**) Cassava stem showing yellow little leaves, short internodes, sprout along the stem and axillar proliferation. (**C**) Longitudinal cut of a healthy cassava stem. Black circles indicate normal distance among internodes. (**D**) Longitudinal cut of diseased cassava stem. Red circles indicate shorter distances among internodes. (**E**) Cross section of a healthy cassava stems (**left**) and a CWBD-affected plant showing vascular discoloration (**right**).

The characteristic symptoms of CWBD include dwarfism and proliferation of weak, spindly sprouts on the stakes (Figure 1). Cassava stems then develop short internodes, with small yellowish leaves, and show dark vascular necrosis, while the roots are thinner, and smaller (Figure 2). Attempts to transmit the disease via grafting have also been challenging. When using grafts from unaffected parts of the plant, the disease could not be transmitted, and when using grafts from affected stems, the observed vascular necrosis kills most grafts used in screenhouse assays (not shown). Due to the significant effect that CWBD has on stem development, cassava vegetative seed production (stakes) is significantly limited, forcing farmers to acquire planting material from other sources and therefore increasing the risk of introducing additional seed-borne pathogens [4,5].

Initial efforts to identify the causal agent of CWBD in SEA were targeted toward phytoplasma specifically belonging to the 16SrI ribosomal group, as suggested by the first report of a witches’ broom disease of cassava in the south pacific islands of Wallis and Futuna in 2004 [10]. Indeed, since 2013, this pathogen has been detected in affected plants, first in Vietnam [7], and then in Cambodia, Laos and Thailand [4,8,9]. Recent surveys carried out in 2020 by national plant protection officers confirm the current presence of the disease in the region (Supplementary Table S1), but lack information on the identity of phytoplasma in affected plants.

Phytoplasmas are phloem-limited bacteria belonging to the class Mollicutes (Acholeplasmataceae) that lack a cell wall and require sap-feeding hemipteran insect vectors for their dispersal [11]. Naturally, leafhoppers (Cicadellidae), planthoppers (Fulgoromorpha) and psyllids (Psyllidae) can transmit phytoplasma [12,13]. Phytoplasmas are classified either as a ‘*Candidatus* phytoplasma’ species, based on percent sequence identity and according to the phylogenetic relationship of their 16S rDNA sequences [14], or as ribosomal groups and subgroups based on restriction fragment length polymorphism (RFLP) analysis of the same ribosomal region [15,16,17]. According to this system, there are 45 ‘*Candidatus* phytoplasma’ species distributed in 33 ribosomal groups [18] and phytoplasma of up to four different ribosomal groups has been reported in CWBD-affected plants from SEA (See below).

In general, the symptoms of witches’ broom are associated with phytoplasma infections and have been reported in more than 116 plant species [19], making phytoplasma an emergent group of crop pathogens [20,21]. Nevertheless, phytoplasma detected in cassava in the Americas (ribosomal group 16SrIII) is found in plants with different symptoms such as a frogskin-like appearance of the roots [22,23,24], or a proliferation of shoots starting at the base of the main stem [25,26]. Current available protocols designed to detect CWBD phytoplasma [27] give unspecific and inconsistent results (not shown), and, to the best of our knowledge, there are no association studies of any of the reported phytoplasma and witches’ broom symptoms in cassava. Although such results can be a consequence of specificities of phytoplasma biology (e.g., tropism, low titers, and inability to grow in vitro), it is important to consider that fungal and viral pathogens have been also reported to induce witches’ broom-like symptoms in plants. *Moniliophthora perniciosa* causes witches’ broom disease in cacao (*Theobroma cacao*). The top part of infected cacao plants grows a green broom which dries up as the fungal infection progresses [28]. In several ornamental cherry species, branches of plants infected with *Taphrina weisneri* swell and shoots with many smaller and thicker leaves emerge at the point of swelling [29]. In Longan, a potyvirus (Longan witches’ broom-associated virus) is associated with a characteristic dense shoot on branches of infected plants [30,31]. Considering that cassava is a vegetatively propagated crop, i.e., prone to build up mixed pathogen infections, further research on the causal agent of CWBD should also look further than phytoplasma. 

Understanding the etiology of this disease is critical for managing its impact. Since the first confirmation of phytoplasmas in diseased plants more than fifty years ago [32], diagnostic assays have been developed to identify and characterize them. As reviewed in [18], commonly used techniques include DNA pattern analysis using restriction enzymes, conventional and quantitative polymerase chain reaction (PCR)-based assays, microarrays, enzyme-linked immunosorbent assays, and loop-mediated isothermal amplification. Among these methods, PCR-based techniques are most preferred because of their high sensitivity and specificity, considering that phytoplasmal DNA transcripts accumulate poorly in infected tissues and can be unevenly distributed in the entire plant [33,34]. Phytoplasma detection via PCR primarily hinges on the amplification of various highly conserved bacterial genes, including the 16S-23S ribosomal DNA spacer region [35,36,37], rp [38], secY [39], secA [35,40], elongation factor TufB [40] and chaperon GroEL [41], among other genes.

Several research groups have successfully characterized phytoplasma strains across plant species using these housekeeping genes. For instance, using a combination of nested PCR and RFLP, based on the 16S rDNA gene, refs. [22,24] characterized phytoplasma associated with CWB-like symptoms in Vietnam and Brazil, respectively. In another study, real-time PCR amplifying the 16S rDNA was used to monitor *in planta* spread and colonization of *Candidatus* Phytoplasma aurantifolia in lime plants [42]. A similar approach was recently used to quantify the intercellular accumulation of *Candidatus* Phytoplasma solani in different parts of infected tomato plants [43]. Scanning different parts of infected cassava plant using quantitative PCR could provide deep insights on localization and accumulation of phytoplasma in cassava, which is currently unknown. It is worth noting that though PCR-based procedures dominate most phytoplasma characterization studies, their success is impeded by several constraints. These include costly laboratory infrastructure, the need for highly trained personnel to extract high-quality DNA and run lengthy downstream analytics, and uneven and low concentration of phytoplasmal DNA in infected plant tissues, a typical case in most susceptible plant species. In general, for CWBD-phytoplasma detection, the universal phytoplasma primer pair P1A/P7A [44] and double-nested PCR have been widely used [7,8,9]. These amplify a 1800 bp fragment of the 16S rDNA region (16S-23S spacer region plus a portion of the 5’ region of 23S rRNA gene). After a first round of PCR, the primer pair R16F2N/R16R2 [45] is used to amplify a fragment of 1200 bp. Next, a second nested PCR using primers R16(I)F1 and R16(I)R1 [46] is employed, which yields a 1100 bp product. In our experience, this protocol has not been efficient in detecting the reported CWBD phytoplasma sequences. A high percentage of false positives is obtained from symptomatic plants, as verified via Sanger sequencing of the PCR bands (Supplementary Figure S1).

## 3. CWBD in the Field

**Figure 3:**
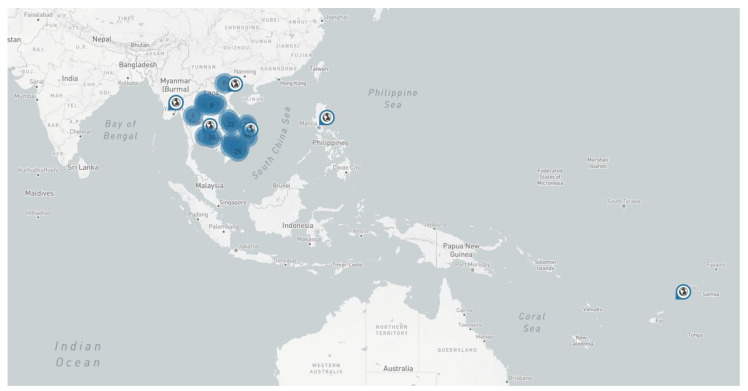
Map showing the current geographical location of CWBD symptoms reported in Asia, according to a global literature search using Google Scholar. Information from CIAT’s annual reports is included. Locations reporting the disease more than once are indicated with a number in a blue circle, otherwise the symbol refers to a single report of the disease. Links to each report are available from the interactive map at: https://pestdisplace.org/embed/news/map/disease/3 (accessed on 15 May 2023) [47]. This information was last updated on 30 March 2023.

Specific literature searches for CWBD reports and extensive routine surveys conducted by CIAT cassava program and local partners across the SEA region [7,8,9] allow us to map the occurrence of CWBD since its first report in 2005 (Figure 3). Most recently, surveys were organized using standard protocols applied at a regional scale, in cassava fields 3–6 months after planting, in order to carry out comparative analyses [4,5]. To visualize and communicate the results among partners in different countries, the data were uploaded onto the PestDisPlace platform [47] and incidence levels calculated as a percentage of symptomatic plants per field per province [5,48]. During our last survey in 2020, CWBD was reported in Cambodia, Laos and Vietnam (Figure 1). The characteristic external symptoms in the aerial part of the cassava plants coincided with previous descriptions in all locations (Figure 2). It is interesting that, in any case, we did observe proliferation of shoots starting at the base of the main stems, which is a characteristic of cassava ‘witches’ broom’ reported in Argentina and Brazil [25,26]. 

A previous regional survey carried out in 2014 on a total of 126 cassava fields in Laos, 99 in Cambodia and 141 in Vietnam recorded CWBD incidences > 40% at the province level [4]. These incidences were comparable to those reported in 2010–2012, and, since 2015, reports of cassava mosaic disease (CMD) caused by the begomovirus Sri Lankan cassava mosaic virus have taken over disease surveillance activities in this region [5,48,49]. Recent national reports show CMD incidences of more than 80% at the province level and confirmed the lurking presence of CWBD in the region PDR (Figure 3; Supplementary Table S1). 

In the field, symptoms of CWBD in SEA are readily recognized along the main stems of a cassava plant, and, in cases of low incidences, this allows for an efficient positive selection by farmers at harvest time. Nevertheless, compared to CMD, CWBD symptoms develop later in the cassava crop cycle, so that stems collected from apparent asymptomatic plants will enter the next crop cycle. Recent field trials in Cambodia show that for two local popular varieties, Rayong 5 and KU50, the latter showing tolerance to CMD [50], symptoms of CWBD appear months later than those of CMD, during the dry period of the season, even when starting with infected planting material (Table 1). A high incidence of cassava stems affected by CWBD will impact the amount and quality of stems available for the next season, pushing farmers to look for alternative sources of planting material.

Noticeably, CWBD symptoms are irregularly expressed on branches from the same plant (Figure 1B), and besides dwarfism and yellowing of young leaves, vascular necrosis has been reported in all cases. Vascular pathogens such as phytoplasmas trigger Ca^2+^ influx, leading to sieve-element occlusion and abnormal callose deposition followed by sieve-element necrosis [51,52]. In the case of witches’ broom-like symptoms caused by fungi, the best studied example is probably that of *M. perniciosa*, where production of gibberellic acid is associated with the abnormal leaf development [28]. Recently, it has been shown that in the case of phytoplasma, several of these symptoms can be triggered by the protein effector SAP05. These effectors simultaneously prolong the host lifespan and induce witches’ broom-like proliferations [53]. Surveys have shown that stem symptoms of CWBD were identical in Vietnam, Cambodia and Laos, pointing to a common disease-causing mechanism occurring in the whole region.

## 4. Phytoplasma Sequences Detected in Cassava Plants 

**Figure 4:**
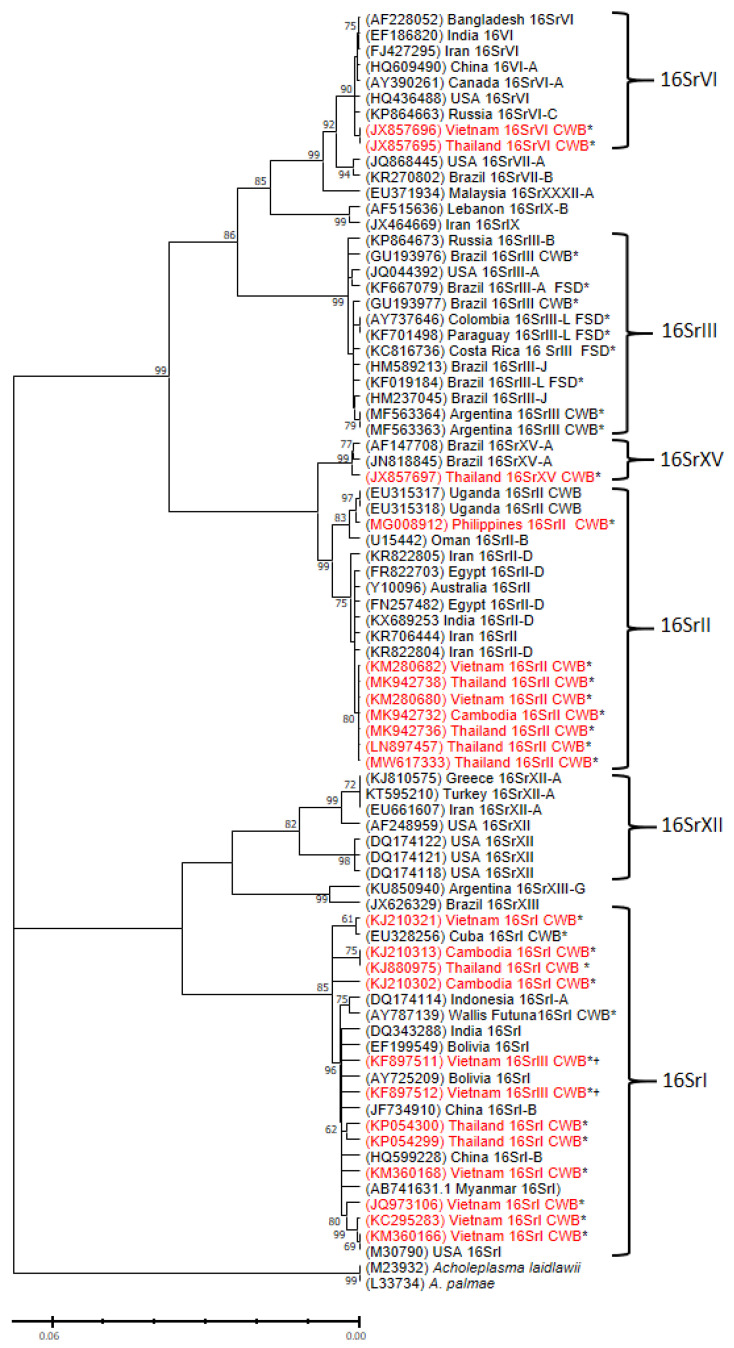
Phylogenetic tree inferred from analysis of 16S rDNA sequences from phytoplasma showing the relationships among representatives of phytoplasma strains infecting vegetables and the ones associated with CWBD in SEA. The tree was constructed using the maximum-likelihood method and Tamura 3-parameter model [56]. Branch lengths are measured in the relative number of substitutions per site; numbers on the branches are bootstrap values for 1000 replicates (only values ≥ 60% are shown) and the scale bar represents the number of nucleotide substitutions per site. GenBank accession numbers for each taxon are given in parentheses. Phytoplasma sequences detected in cassava are indicated with an asterisk (*) and those from cassava in SEA are indicated in red. A cross (†) indicates those sequences that appear misannotated as 16SrIII in Genbank. *Acholeplasma palmae* and *A. laidlawii* were used as out-group. The corresponding 16S rDNA group is indicated to the right.

Phytoplasmas infecting cassava were first reported in the Americas in plants affected with cassava frogskin disease (CFSD), where the roots of infected plants develop a rugose peel that resembles the skin of lizards or frogs, which is accompanied by a significant and gradual decrease in root yield over several crop cycles [23]. Unlike CWBD, little or no symptoms are observed in the upper part of a plant with CFSD and sequence analysis of the 16S rDNA region, group all CFSD-associated phytoplasmas within the 16SrIII group (Figure 4). Interestingly, a couple of reports describe a 16SrIII phytoplasma in plants with “witches’ broom”-like symptoms in cassava in Brazil and Argentina; nevertheless, the photographic records show that witches’ broom-like symptoms observed in the Americas refer to different symptoms, characterized by an increased branching that starts at the base of the main stem of the cassava plant [25,26]. No such symptoms, or phytoplasma of the 16SrIII group, have been observed in plants with CWBD in SEA.

Phytoplasma sequences reported from plants affected by CWBD can be found under different descriptive names: 1: Cassava witches’-broom aster yellows phytoplasma; 2: Cassava witches’-broom disease phytoplasma; 3: ‘*Manihot esculenta*’ witches’-broom phytoplasma (Thailand); 4: ‘*Manihot esculenta*’ dwarfism and witches’-broom phytoplasma; 5: *Candidatus* phytoplasma aurantifolia; 6: ‘*Manihot esculenta*’ witches-broom phytoplasma; and 7: Vietnamese cassava phytoplasma. This shows a lack of consensus among different groups studying the same disease. To organize the sequence information, the entries were curated for length, redundancy and incomplete annotation. Considering that the smallest standard nested PCR tests to detect phytoplasma amplify sequences of ~800 bp (second nested PCR) and classification of phytoplasma 16S rDNA sequences at the sub-group level require at least ~1300 bp [54], we selected sequences in this range to continue with the analysis. In total, 85 CWBD-related 16S rDNA sequences were included (Supplementary Table S1). Phytoplasma reference sequences for each group [11] were also included in the analysis (Figure 4).

Phytoplasma sequences detected in CWBD-affected cassava in SEA reported to date fall into at least four subgroups: 16SrI, 16SrII, 16SrVI and 16SrXV (Figure 4), suggesting independent incursions of phytoplasma in cassava and likely the presence of multiple alternative hosts; however, none of these sequences are related to experimental studies associating them with the symptoms of CWBD nor they confirm phytoplasma as a causal agent of the disease. We found an additional CWBD-phytoplasma sequence from Vietnam annotated as belonging to the 16SrIII subgroup (Genbank no. KF897511), but our analysis clearly shows this is a misannotation of a sequence that actually corresponds to the 16SrI group (Figure 4). 

Interestingly a 16SrII phytoplasma sequence was detected in cassava plants with CWBD in Kawanda, Uganda ([55]; Genbank no. EU315317). The symptoms described in these plants are similar to those observed in SEA and the sequence shared closer similarities with accession MG008912 from the Philippines (Figure 4). This finding may suggest a possible introduction of infected material to Africa; however, there have been no other reports of the disease from Uganda or anywhere else in Africa since 2009.

## 5. Conclusions and Future Insights

CWBD, largely unnoticed in SEA before 2010, emerged soon after as a major disease affecting cassava production in this region, and current surveillance data show the disease is still present in Thailand, Cambodia, Vietnam and Laos (Figure 3). Although the biology and epidemiology of witches’ broom symptoms is, in most cases, associated with phytoplasma infection, not much progress has been made to confirm phytoplasma as the causal agent of CWBD (no pathogenicity tests nor field association studies could be found in the literature); therefore, it remains a mystery. As mentioned before, chip-bud grafting, a standard method used in cassava to test transmissibility of disease symptoms, is inefficient due to the vascular necrosis observed in all CWBD-affected plants (Figure 2); consequently, no study on the transmissibility of CWBD via grafting has yet been reported. 

Furthermore, our efforts to systematically analyze and detect the occurrence of phytoplasma in symptomatic and/or grafted plants using published protocols [4,7,8,9,27], have not been successful (Supplementary Figure S1). As a standard, nested PCR protocols are used for phytoplasma detection and characterization, and group identification is based on the second PCR product obtained after two rounds of PCR using ‘universal primers’ [44,45], increasing the risk of obtaining false positives. It is intriguing to observe different genetic populations of phytoplasma infecting cassava reported in SEA versus the Americas (Figure 4). These groups have been reported using a mix of specific and generic detection protocols so that the resulting difference is less likely due to the use of a biased detection method. It is not yet known whether the detection of multiple phytoplasma in the region could also reflect past rounds of vector infestations (leafhoppers, planthoppers and psyllids) in cassava fields not necessarily associated with CWBD. There is an urgent need to develop reliable molecular diagnostics tests for CWBD in the region. Limited knowledge about the distribution of the causal agent of CWBD within the plant and its transmission in the field has hindered efforts to study its pathogenicity and diagnostics. Our greenhouse observations show that when propagating stakes from affected plants, symptoms will appear only from stakes taken from affected parts of the plant. Grafting tests have also shown that grafts taken from parts of the cassava stem that do not show symptoms are not effective in transmitting the disease to susceptible varieties such as KU50. On a related note, although KU50 has shown tolerance to CMD [50], the observed susceptibility of this variety to CWBD (Table 1) should be a matter of concern for CMD disease management. KU50 was released in 1992 and is the most widely planted cassava variety in this region due to its high dry matter and starch content [1,57]; breeding and screening for alternative varieties with resistance to CWBD should be prioritized.

This review also noted that given the detection of phytoplasma of different ribosomal groups in SEA, mixed infection is likely to occur in a single cassava plant, as reported in other systems [58,59]. Whether mixed infections affect the detection of specific phytoplasma groups and whether they are associated with differences in disease severity is still unknown. We should also note that focusing on phytoplasma as a causal agent could mask the occurrence of other pathogens, such as those indicated in the text that may be associated with witches’ broom symptoms.

As mentioned above, grafting experiments suggest that the causal agent is localized, or at least in higher concentrations, in specific parts of the plant. This observation should be considered when validating field surveillance observations, where, for practical reasons, only young leaves are collected for further diagnostic tests. Another factor is the relative longer time that CWBD symptoms take to fully develop in the field (Table 1), impeding the early detection of contaminated planting material. The need for solid diagnostic tests, which require the evaluation of a range of symptomatic plants using standard sampling protocols, cannot be over emphasized. Further insights about the disease require the availability of multiyear trials in insect-proof facilities (e.g., screen houses), a limiting factor in SEA. 

The co-occurrence of CMD in the region [5,48], the possibility of mixed pathogen infections and/or alternative CWBD-associated pathogens [29,30,31] and informal cross-border seed exchange networks in SEA [6] add to the cassava disease burden in this region. These conditions favor the development of negative synergisms and confound diagnostics, making it complex to implement any integrated disease management strategies. It is, therefore, critical to characterize CWBD under controlled conditions at a molecular level and develop rapid diagnostic tests. This would help identify and tackle the transmission route of CWBD, the effect of farming practices such as varietal switching between seasons and varietal mixing on CWBD pressure, and identify resistant genotypes and potential symptomless reservoirs of CWBD by isolating and testing DNA from weeds, grasses and other vegetatively propagated crops within and in the peripheral of cassava fields.

## Figures and Tables

**Table 1:** Symptoms incidence (%) at different time points using planting material from CMD and CWBD-affected plants in comparison to positive selected (no symptoms of disease) planting material. More than 95% of plants coming from CMD-affected planting material showed symptoms at 22 days after planting (d.a.p.) (numbers in red), while those coming from CWBD-affected plants did not show any symptom at this time point (numbers in blue). Values in parentheses indicate standard error of the mean values.

Variety	Seed Type ^1^	22 d.a.p.		66 d.a.p.		158 d.a.p.	
		CMD	CWBD	CMD	CWBD	CMD	CWBD
KU50	+selection	2.1 (2.1)	0	6.3 (3.9)	8.3 (8.3)	12.4 (4.0)	24.5 (8.3)
	CMD	100 (0)	0	75 (25)	2.1 (2.1)	100 (0)	27.1 (2.1)
	CWBD	20.8 (2.4)	0	31.2 (7.1)	12.5 (7.2)	40.8 (4)	29.2 (7.2)
Rayong 5	+selection	16.7 (0)	0	27.1 (3.9)	0	39.6 (4)	37.5 (0)
	CMD	97.9 (2.1)	0	100 (0)	0	100 (0)	8.3 (0)
	CWBD	56.3 (3.9)	0	66.7 (14.2)	4.2 (2.4)	77.1 (5.9)	45.8 (2.4)

^1^ Stakes used for the experiment were positive-selected (+selection), i.e., showing no symptoms of disease, or coming from plants showing symptoms of disease (CMD or CWBD). Twelve plants per seed type x treatment x disease, in four replicates, were used in this assay.

## Data Availability

The data presented in this study in Table 1 are available on request from the corresponding author. Data described in Supplementary Table S2 are available as a public dataset https://doi.org/10.7910/DVN/F8IU7F, accessed on 15 May 2023.

## Supplementary Materials

The following supporting information can be downloaded at: https://www.mdpi.com/article/10.3390/plants12112217/s1, Supplementary Table S1: Field surveys for CWBD in Southeast Asia; Supplementary Figure S1: Unspecific detection of bacterial sequences using primers for phytoplasma detection.
