# Supplementary material for: Cassava Witches’ Broom Disease in Southeast Asia: A Review of Its Distribution and Associated Symptoms
Source: Plants (Basel). 2023 Jun 4;12(11):2217. doi: 10.3390/plants12112217 (PMC10255860; doi:10.3390/plants12112217)
Supplement: Supplementary file 1 [file plants-12-02217-s001.zip › plants-2228413-supplementary Figure S1.zip!/plants-2228413-supplementary Figure S1.pdf]

A

Phenotype	PCR (-)	PCR (+)	Total
**Healthy**	427	17	444
**Classic witches' broom symptoms**	12	22	34
**Total**	439	39	478

B

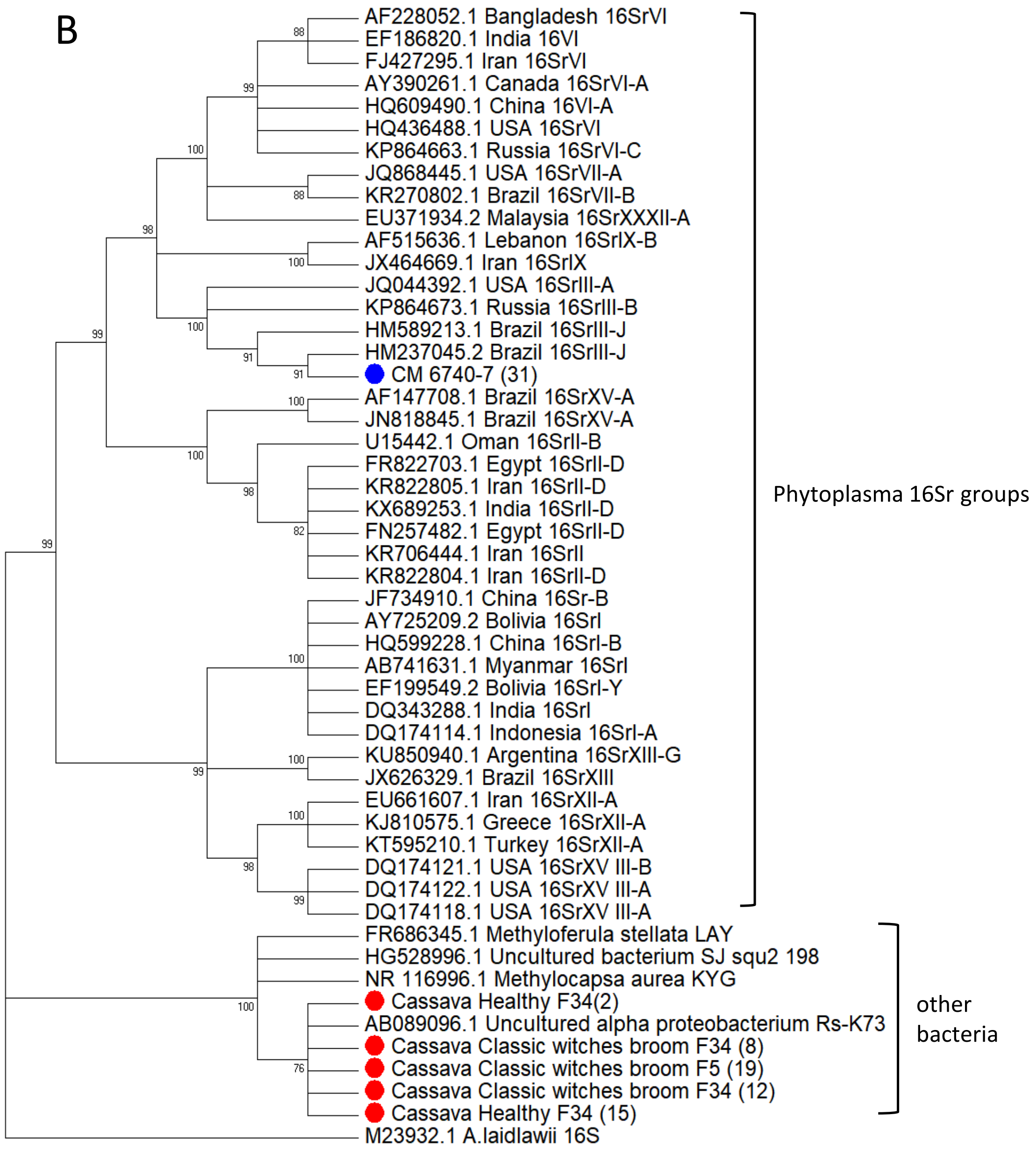

Supplementary Figure S1. Unspecific detection of bacterial sequences using primers for phytoplasma detection. A. 478 plants collected from affected fields were evaluated by PCR for phytoplasma infection using generic primers P1A/P7A and R16F2N/R16R2 [15]. PCR products of the expected size and obtained from healthy and CWBD-affected plants were sequenced (red dots in **B**). These sequences shared 97-99% nucleotide identity with sequences of *Alphaproteobacteria*, *Methyloferula stellata*, and *Methylocapsa aurea* using BLAST analysis. **B.** Evolutionary relationships of sequences obtained in **A** (false positives) with 16S phytoplasma groups. The tree was inferred using the Neighbor-Joining method available in MEGA7 using a bootstrap of 1000x replicates. Branches with bootstrap values below 75% are collapsed. A cassava infected with phytoplasma of the 16SrIII group (genotype CM6740-7) was used as an amplification control and its corresponding sequence is indicated in the tree with a blue dot.
