# Supplementary material for: Cassava Witches’ Broom Disease in Southeast Asia: A Review of Its Distribution and Associated Symptoms
Source: Plants (Basel). 2023 Jun 4;12(11):2217. doi: 10.3390/plants12112217 (PMC10255860; doi:10.3390/plants12112217)
Supplement: Supplementary file 1 [file plants-12-02217-s001.zip › plants-2228413-Supplementary_Table_S1.zip!/plants-2228413-Supplementary_Table_S1.pdf]

Supplementary Table S1. **Field surveys for CWBD in Southeast Asia**. Incidence of CWBD in Vietnam, Lao PDR and Cambodia recorded in 2014 [4] and during routine surveys in 2020 by national plant protection officers in Vietnam, Lao PDR and Cambodia. One every fourth plant (60 plants per field) was inspected and photographed following an X pattern, covering an area of 1 ha. % incidence indicates the average number of symptomatic plants per field per province in the sample (60 plants). 0 = none of the plants surveyed showed CWBD symptoms in any of the surveyed fields. The number next to province names indicates the number of fields inspected in each province.

Country	Region	Province (# fields) 2014	% Incidence (SE) 2014	Province (# fields) 2020	% Incidence (SE) 2020
Vietnam	North	-	-	Bac Giang (5)	0
		-	-	Dien Bien (4)	0
		-	-	Hoa Binh (3)	0
		-	-	Lai Chau (4)	0
		-	-	Lang Son (4)	0
		-	-	Lao Cai (4)	0
		-	-	Nghe An (12)	0
		-	-	Phu Tho (5)	0
		-	-	Son La (16)	0
		-	-	Thai Nguyen (4)	0
		-	-	Thanh Hoa (8)	0
		-	-	Tuyen Quang (4)	0
		-	-	Yen Bai (12)	0
	Center			Binh Dinh (4)	0
		Đăk Lăk (20)	15.00	Đăk Lăk (5)	0
		-	-	Gia Lai (36)	0.046
		-	-	Kon Tum (3)	0
		Phú Yên (19)	27.053	Phú Yên (11)	0
		-	-	Quảng Bình (4)	0
		-	-	Quảng Nam (8)	1.875
		Quang Ngai (20)	27.31	Quang Ngai (12)	0
		-	-	Quảng Trị (8)	0
		-	-	Thừa Thiên Huế (4)	0
	South	Ba Ria – Vung Tau (20)	12.5	Ba Ria – Vung Tau (4)	0
		-	-	Binh Duong (4)	2.083
		Binh Phuoc (21)	1.24	Binh Phuoc (12)	0
		-	-	Bình Thuận (20)	0
		-	-	Đak Nông (8)	0
		Dong Nai (20)	14.8	Dong Nai (5)	0
		-	-	Khánh Hòa (5)	0
		-	-	Phu My (4)	0
		Tay Ninh (21)	0.7	Tay Ninh (28)	0
Lao PDR	North	-	-	LaoungNamTha (2)	0
		Saiyabouly (20)	17.0	Saiyabouly (8)	0
		Vientiane (22)	12.0	Vientiane (3)	0
		Xiangkhouang (6)	8.67	-	-
		-	-	Xaysomboun (3)	6.111
	Center	Bolikhamsai (24)	25.79	Bolikhamsai (11)	2.879
		-	-	Khammoun (1)	1.667
	South	Champasack (25)	28.24	Champasack (24)	0.625
		Salavan (25)	9.6	Salavan (7)	5.952
		-	-	Sekong (1)	3.448
		-	-	Attapue (6)	0.833
Cambodia	North	-	-	Preah Vihear (4)	0
		Banteay Meanchey (20)	1.2	Banteay Meanchey (24)	0
		-	-	Oddor Meanchey (16)	0
		-	-	Rattanakiri (4)	0
		-	-	Steung Treng (4)	0
	Center	-	-	Siem Reap (8)	0
		Battambang (20)	32.3	Battambang (26)	0
		Pailin (19)	38.42	Pailin (10)	0.167
		-	-	Kampong Thom (12)	0
		Kratie (20)	56.3	Kratie (18)	0.093
		-	-	Mondulkiri (4)	0
	South	Kampong Cham (20)	54.3	Kampong Cham (4)	0
		-	-	Pursat (4)	0
		-	-	Tboung Khmum (12)	0.139
